# Development of a fish oil–nanoemulsion gel as a drug-delivery system to prevent capsular contracture

**DOI:** 10.1038/s41598-024-81122-6

**Published:** 2024-11-26

**Authors:** Mohuya Paul, Sang Gue Kang, Jungkyun Im, Woo Jin Song

**Affiliations:** 1 Department of Electronic Materials, Devices, and Equipment Engineering, Soonchunhyang University, Asan, 31538 Republic of Korea; 2 Department of Plastic and Reconstructive Surgery, Soonchunhyang University College of Medicine, Seoul, 04401 Republic of Korea; 3 Department of Chemical Engineering, Soonchunhyang University, Asan, 31538 Republic of Korea

**Keywords:** Fish oil, Nanoemulsion, Gel, Omega-3, Capsular contracture, Breast cancer, Nanoparticles, Drug delivery, Implants

## Abstract

Capsular contracture, a significant complication of breast-implant surgery, causes pain and deformation following the formation of a tight capsule around an implant. Current surgical and non-surgical treatment options are invasive, expensive, and typically administered for prolonged periods, potentially leading to side effects. To address these challenges, we developed a novel fish oil-encapsulated, poloxamer-based nanoemulsion gel with omega-3 (ω3) as the primary active component (NE-ω3 gel; N3G). This formulation can be injected during surgery, reducing the need for prolonged administration of medications and minimizing associated side effects. N3G was prepared through a two-step process involving the emulsification of fish oil followed by gelation with poloxamer to create a thermoreversible gel. Dynamic light-scattering analysis confirmed a uniform distribution of nanoemulsion particles, which had a mean diameter of 287 ± 8.599 nm and a PDI of 0.29 ± 0.047. FTIR and DSC analyses validated the encapsulation of fish oil within poloxamer micelles. Drug-release analysis demonstrated that more than 96% of fish oil was released within 10 h. In in vivo experiments, rats (*n* = 30) were divided into three groups: a negative control group (G1), a positive control group receiving fish oil (G2), and an experimental group with implants coated with N3G (G3). G3 exhibited a 21.2% reduction in capsular thickness compared to G1 and a 5.6% reduction compared to G2. In addition, significant decreases in fibrosis and myofibroblast counts were observed in G3. These results, supported by histological and gene expression analyses, highlight the effectiveness of N3G in reducing inflammation and fibrosis. This study underscores the therapeutic potential of N3G for capsular contracture, based on its persistence, thermoreversibility, and improved dosing and storage characteristics.

**Supplementary Information:**

The online version contains supplementary material available at 10.1038/s41598-024-81122-6.

## Introduction

Capsular contracture is a major challenge in breast-implant surgery involving cosmetic augmentation and reconstruction. Following the formation of a tight capsule around breast implants, capsular contracture results in firmness, deformation, and pain. It arises from a foreign body response and is closely associated with inflammation and immune responses that promote fibroblast and collagen growth^1,2^. Capsular contracture occurs with a prevalence of 19–25% in reconstruction surgeries, which increases to 40% when coupled with radiation therapy^3,4^. Breast cancer accounts for 24.5% of all female cancer cases, making it the most common cancer affecting women globally. Moreover, the number of individuals undergoing breast reconstruction surgery is gradually increasing. With implant breast reconstruction used in over 70–80% of cases, the demand for effective strategies to prevent capsular contracture is also rising^5^.

Surgical treatment for capsular contracture is effective but risky and expensive, prompting interest in drugs such as pirfenidone, leukotriene receptor antagonists (LTRAs) such as zafirlukast and montelukast, and steroids such as triamcinolone. LTRAs are promising for the early prevention of capsular contracture but cause headaches and in rare cases hepatic failure. These drugs are not typically recommended for patients with cancer undergoing breast implantation^6–13^.

Altering the surface of silicone implants can lower the likelihood of capsular contracture^2,14–16^. A variety of strategies have been developed in the past to mitigate capsular contracture, including the use of triamcinolone-coated silicone implants, cyclodextrin polymer-coated implants, electrospun zafirlukast-loaded fiber mats, paclitaxel-loaded thermogels, and thermosensitive chitosan-based hydrogels^14,17–22^. These methods are effective, but they have certain disadvantages. For instance, triamcinolone may cause local skin and muscle thinning, delayed wound healing, and adrenal suppression^22^; cyclodextrins can lead to nephrotoxicity and have limited solubility^22^; zafirlukast may result in headache, nausea, and potential hepatic dysfunction^10^; paclitaxel is associated with neutropenia, alopecia, and peripheral neuropathy^23^; and chitosan can trigger allergic reactions^24^. Hence, these techniques do not offer a complete solution to capsular contracture, making room for improvement.

The complex origins of capsular contracture, involving inflammatory cytokines such as tumor necrosis factor-α, transforming growth factor (TGF)-β, and interleukins (ILs), have prompted the search for even more effective preventive strategies^1^. One promising approach is the use of omega-3 (ω3) polyunsaturated fatty acids (PUFAs), particularly eicosapentaenoic acid (EPA) and docosahexaenoic acid (DHA), from fish oils. ω3 PUFAs, which have anti-inflammatory and antifibrotic properties, benefit the heart, liver, and kidney^25,26^. They modulate the arachidonic acid (AA) pathway by competing with AA and reducing the levels of proinflammatory eicosanoids^27^. EPA, which is processed through the cyclooxygenase (COX) and lipoxygenase (LOX) pathways, reduces the levels of inflammatory products^28^. Moreover, ω3 promotes the formation of pro-resolution factors, such as resolvins, promoting inflammation resolution and healing^29,30^. Supplementation with ω3 can prevent capsular contracture by inhibiting the TGF-β pathway, thereby reducing collagen deposition and inflammation^31–33^. Although ω3 PUFAs are generally safe in humans, they have several limitations. Despite their potential to reduce capsular contracture, their practical implementation faces substantial challenges. The bioavailability and poor aqueous solubility of ω3 PUFAs, particularly in fish oil supplements, can hamper absorption and lead to gastrointestinal side effects. Moreover, the oxidation of fish oil not only produces an unpleasant odor but also complicates its storage, thereby making it less practical for long-term use. Thus, a suitable drug-delivery system (DDS) is required to enhance the bioavailability and therapeutic efficacy of ω3 PUFA supplements.

In this study, we have formulated fish oil-encapsulated nanoemulsion gel (NE-ω3 gel; hereafter, N3G), which showed anti-inflammatory properties due to ω3 PUFAs. Nanoemulsions are nanosized colloidal dispersions consisting of two immiscible phases of oil and water. While the oil phase encapsulates the fish oil, improving its bioavailability and, therefore, therapeutic efficacy, the water phase facilitates better absorption in the biological system, providing improved drug delivery applications. Additionally, the use of non-toxic precursors in the formulation makes it a biocompatible and potentially low-risk approach. N3G leverages the anti-inflammatory properties of ω3 fatty acids, providing significant therapeutic benefits without the adverse effects commonly linked to synthetic drugs. Thermoreversibility was introduced in this formulation by using poloxamers (Pluronics), which have excellent injectability and show sustainable release of encapsulated drugs, reducing the drug doses needed^34,35^. N3G was formulated, characterized, and assessed for its ability to prevent capsular contracture during breast implant surgery using a rat model. The results showed that the formulation has the potential to considerably improve the outcomes of cosmetic and reconstructive breast surgeries.

## Materials and methods

### Materials

Pluronic F-68 (PF68) was obtained from Alfa Aesar (now part of Thermo Fisher Scientific, Korea). Menhaden fish oil, ethyl acetate (EA), polysorbate (TWEEN 80), sorbitan oleate (SPAN 80), polyethylene glycol (PEG)-400, and Pluronic F-127 (PF127) were purchased from Sigma-Aldrich Co. (St. Louis, MO, USA). Deionized (DI) water was used to prepare nanoemulsions.

### Preparation of N3G

N3G was prepared using a two-step approach modified from a reported protocol^36^. In brief, 1 g (13.8 wt%) fish oil was placed in a vial, to which was added 0.8 g TWEEN 80 (11.03 wt%) and 0.2 g SPAN 80 (2.75 wt%) at a 4:1 ratio as a non-ionic surfactant mixture. The non-ionic surfactant ratio was determined by evaluating the effects of different ratios of TWEEN 80 to SPAN 80 on the particle size of the nanoemulsion (Fig. S1). The mixture with the optimum hydrophile-lipophile balance (HLB) for oil-in-water nanoemulsions^37^ was used for emulsification. In addition, 0.25 g PEG 400 (3.4% wt) was added to the mixture as a co-surfactant, and the solution was mixed using a magnetic stirrer (IKA RCT basic, Korea) until it became homogeneous. PEG 400 was added to the oil phase during preparation of the nanoemulsion to further reduce the oil droplet size^38^. Non-ionic surfactants alone cannot reduce the o/w interfacial tension sufficiently to form a stable nanoemulsion^36,39,40^. Due to its hydrophilicity, PEG 400 cooperates with non-ionic surfactants to reduce the interfacial tension and thus enable formation of a nanoemulsion. The amount of PEG 400 was also assessed to determine the optimal quantity required to achieve a sufficiently small oil droplet size in the nanoemulsion (Fig. S2). DI water was added dropwise to the oil phase, and the mixture was stirred for 15 min to produce a nanoemulsion (NE-ω3).

A small amount of nanoemulsion was placed in a vial and diluted with DI water. The poloxamers PF127 (30% w/v) and PF68 (1% w/v) were added to the diluted nanoemulsion as gelling agents to transform the nanoemulsion into a gel-like state. Gelation of the nanoemulsion was initiated using the cold method^41^, because poloxamers are more soluble in cold water. The polymer mixture was added to the nanoemulsion on ice and stirred vigorously. The solution was degassed overnight, yielding bubble-free N3G.

### Particle size, polydispersity index (PDI), and zeta potential analyses using dynamic light scattering (DLS)

The nanoemulsion was analyzed via DLS using a Zetasizer particle size analyzer (Malvern Panalytical Ltd. UK) to determine the average particle size (Z-average [Z_avg_]), PDI, and zeta potential. Sample (0.1 mL) was placed in a vial and diluted 200-fold with DI water. This solution was used to determine the Z_avg_, PDI, and zeta potential of the nanoemulsion. The refractive index (RI) and absorption coefficient for fish oil were set to 1.464 and 0.001, respectively, for particle size and PDI analyses. DI water was used as the dispersant. For zeta potential measurement, a disposable clear zeta capillary cell (DTS1070, Malvern Zetasizer) was used. The cell was thoroughly flushed with ethanol and DI water, filled with sample solution, and fitted with the stopper and thermal plates. The experiments were repeated at least three times to ensure the consistency and reliability of the results.

### Effects of temperature and pH on NE-ω3 and N3G

Fish oil is prone to oxidation, even at room temperature. A preliminary stability study was conducted on the nanoemulsion formulation before evaluating its behavior at elevated temperature and pH. The formulation (NE-ω3) was assessed in terms of changes in the Z_avg_, PDI, and zeta potential over 24 h. The sample was incubated at room temperature and analyzed at hourly intervals beginning at 0 h (immediately after preparation of the nanoemulsion) via DLS (Malvern Zetasizer instrument, Korea).

To evaluate the effect of temperature, freshly prepared NE-ω3 was incubated in a water bath at 37 °C for 24 h. As a control, a similar formulation was kept at room temperature. After 24 h, the particle size, PDI, and zeta potential were measured via DLS. To investigate the effect of pH on these parameters, NE-ω3 was acidified to pH 5 using 0.1 N HCl solution. The effect of basic pH on NE-ω3 was evaluated by altering its pH to 9 using 0.1 N NaOH solution. In the control, the pH was maintained at 7, the value of healthy breast tissue. Three nanoemulsion samples were individually monitored for changes in particle size, PDI, and zeta potential.

The effects of heat and pH on N3G were assessed via FTIR. N3G was heated at 50 °C for 24 h to assess the effect of temperature. To evaluate the effects of harsh pH conditions, N3G was treated with an acidic or basic solution and incubated for 24 h at room temperature. N3G samples were analyzed by FTIR, and their spectra were compared.

### Fourier transform infrared spectroscopy (FTIR)

FTIR was carried out using a JASCO FT/IR-4600 spectrometer in attenuated total reflectance (ATR) mode with a frequency range of 4000–500 cm^–1^. The FTIR spectra of the NE-ω3-gel and the starting materials (fish oil and poloxamers) were evaluated. A small amount of sample was mounted on the sample stage of the ATR. To assess the FTIR spectra of fish oil and N3G, about 0.1 mg sample was spread over the ATR crystal using a syringe. For poloxamers, 3–4 mg solids were placed over the ATR stage covering the crystal and pressed using the pressure device. Constant pressure was applied to the samples, and each was scanned 32 times in triplicate at room temperature.

### Differential scanning calorimetry (DSC) analysis

N3G and the starting materials (fish oil, PF127, and PF68) were thermally characterized via DSC analysis (TA Q20 series thermal analyzer, TA instruments, Korea). For each experiment, 4 mg sample was weighed, sealed in an aluminum pan, and heated from 35 to 90 °C at a rate of 10 °C/min under a constant nitrogen flow of 50 mL/min. An empty aluminum pan was used as the reference.

### In vitro drug-release assessment

The drug release pattern of N3G was assessed using the membrane-free dialysis method modifying a previously reported protocol^42^. DI water was used as the release medium, to which 10 mL EA was added to dissolve any released fish oil, resulting in a visible layer between the two solvents. Since fish oil is not miscible with water, EA was used as the second solvent because it shows good miscibility with fish oil and is less volatile than other common organic solvents such as hexane and diethyl ether. To cover the surface with N3G, a silicone mini-implant was immersed in N3G solution at room temperature. The process was repeated multiple times until a visible coat of N3G formed on the surface of the mini-implant. Next, the mini-implant was inserted into a perforated tube, which was submerged in release medium and incubated at 37 °C without stirring. Aliquots of 0.5 mL of the EA layer were collected at hourly intervals for 10 h to measure absorbance via UV–vis spectroscopy (JASCO V-730) at a λ_max_ of 263 nm, which was determined beforehand (Fig. S3). The aliquot was returned to the medium after the measurement. Because samples were not diluted, aliquots were added as it is in the medium, maintaining the medium volume throughout the experiment.

### Animal experiment with implants and N3G

The number of rats per group was determined using G*Power software (version 3.1)^43^. A priori power analysis was conducted using the F-test for ANOVA: fixed effects, omnibus, one-way. The parameters were as follows: effect size (f) = 0.60, α error probability = 0.05, power (1–β error probability) = 0.80, and three groups. The analysis yielded a noncentrality parameter (λ) of 10.80, a critical F-value of 3.35, numerator degrees of freedom of 2, and denominator degrees of freedom of 27. The total sample size required to achieve the desired power was calculated to be 30 subjects, with an actual power of 0.800. Therefore, each group consisted of 10 rats to meet the statistical requirements of the study.

Seven-week-old female Wistar Hannover GALAS rats (N = 30; mean weight = 197.8 ± 9.5 g) were obtained from colonies maintained under specific-pathogen-free conditions (Orient Bio, Seongnam, Korea). All animals were housed under standard laboratory conditions at a temperature of 22 ± 2 °C with a relative humidity of 50.0 ± 15.0% (12/12 h light/dark cycle), and they had ad libitum access to food and water. At 8 weeks of age, following the end of the quarantine and acclimatization period, the animals underwent surgery and were subsequently housed in pairs in animal cages after implant surgery. The rats were divided into three groups (n = 10 per group): G1 (negative control), untreated; G2 (positive control), daily treatments of menhaden fish oil via gavage at a dose of 80 µL/250 g (i.e., 297 mg/kg, density 0.93 g/mL at 25 °C) as described previously^31^; and G3 (experimental), received implants coated with N3G by immersion at a dose equivalent to 0.6 g, at which 1 g gel contains 3.02 mg fish oil. The dose (0.6 g N3G per mini-implant) for G3 was determined by evaluating the amount of N3G required to cover both sides of the silicone mini-implant. The rats underwent implantation under general anesthesia with isoflurane, starting in a chamber at 4% followed by maintenance at 1.5–2% on the surgical table. The surgical site was prepared, a 2 cm incision was made in the dorsal lumbosacral region for implant placement under the skin and panniculus carnosus, and the incision was sealed with 4–0 black silk sutures (Ethicon, Inc., Somerville, NJ, USA). Following the experimental procedures, the rats were monitored in terms of behavior, appetite, and health indicators such as weight and toxicity. After 90 days, the rats were euthanized by carbon dioxide inhalation and cervical dislocation by an individual trained and competent in the procedure, and capsular tissues were harvested. Animal care and experimental procedures were authorized and conducted in accordance with the ethics guidelines and approval of the Institutional Animal Care and Use Committee of the Woojung Center for Phenogenomics (Approval No. IACUC2302–012; date of approval: 26 April 2023).

### Histological analysis

Capsule tissue was excised and fixed in 10% neutral buffered formalin. For histological analysis, including hematoxylin and eosin (H&E) and Masson’s trichrome (MT) staining and immunohistochemistry (IHC), serial longitudinal sections of 4 µm thickness were prepared from paraffin-embedded tissue. All capsules from every animal were analyzed, and capsule thickness was consistently measured at the central region to standardize data collection. For IHC, primary antibodies targeting α-smooth muscle actin diluted 1:100 (ab5694; Abcam, Cambridge, UK) were applied overnight at 4 °C. This was followed by development using diaminobenzidine with hematoxylin counterstaining. To minimize sampling bias, measurements were taken in areas where the capsule structure was intact, and representative samples were selected based on consistent criteria, such as similar visual and physical characteristics. Data were collected from various sections of a single specimen, and average values were calculated.

### Quantitative reverse transcription polymerase chain reaction (qRT-PCR)

Gene expression analysis for profibrotic and inflammatory genes (collagen alpha-2(in) chain [COL1A2], TGF-β2, interferon [IFN]-γ, IL-4, IL-6, and IL-10) was performed using qRT-PCR. Tissues were frozen, homogenized in TRIzol, and processed for RNA extraction. Next, RNA was converted into cDNA and analyzed using the CFX384 Real-Time PCR Detection System (Bio-Rad, Hercules, CA, USA). The housekeeping gene β-actin was used to normalize the qRT-PCR gene expression data. The expression levels of β-actin were consistent across all samples, confirming the reliability of normalization and the accuracy of quantification of target gene expression. The primers used for RT-qPCR are listed in Table 1. All samples were tested in duplicate and analyzed for relative gene expression. The cycle threshold values of each group were analyzed using the Livak method^44^.

**Table 1:** Primers used for quantitative real-time polymerase chain reaction.

Target gene	Sequence (5′-3′)
COL1A2	For: GGTGAGCCTGGTCAAACGG
	Rev: ACTGTGTCCTTTCACGCCTTT
TGF-β2	For: ACTGTGTCCTTTCACGCCTTT
	Rev: CCCTGGTACTGTTGTAGATGGA
IFN-γ	For: AAGACAACCAGGCCATCAGCAAC
	Rev: GAACTTGGCGATGCTCATGAATGC
IL-4	For: CAAGGAACACCACGGAGAACGAG
	Rev: CTTCAAGCACGGAGGTACATCACG
IL-6	For: AGGAGTGGCTAAGGACCAAGACC
	Rev: TGCCGAGTAGACCTCATAGTGACC
IL-10	For: GTGAAGACCAGCAAAGGCCA
	Rev: CTCTCGGAGCATGTGGGTCT

### Statistical analysis

Values are means ± standard errors of the mean. Statistical analyses of quantitative data were conducted using StatView software (version 4.51; Abacus Concepts, Berkeley, CA, USA). Analysis of variance (ANOVA) was performed, with Fisher’s protected least significant difference (PLSD) post hoc test when statistical significance was detected. For between group comparisons, p < 0.05 was considered significant. The results of qRT-PCR were normalized to G1 and are expressed as fold-change values.

## Results and discussion

### Preparation and characterization of N3G

**Fig. 1:**
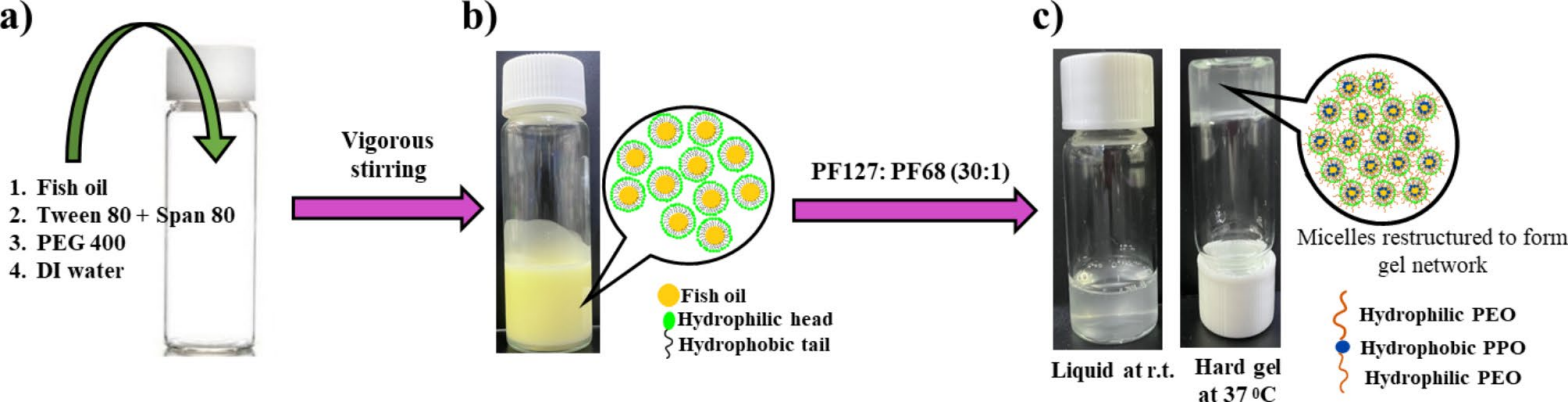
Preparation of NE-ω3-gel (N3G). (**a**) N3G was prepared using a two-step method. The oil phase (fish oil and surfactants) was mixed with DI water. (**b**) The mixture was vigorously stirred to form a nanoemulsion. (**c**) In the second step, a mixture of poloxamers (PF127 and PF68) was added to the diluted nanoemulsion to produce N3G. The final formulation was thermoreversible; it was liquid at room temperature and a gel at 37 °C.

Omega-3 PUFAs, mainly EPA and DHA, present in fish oil have anti-inflammatory and antifibrotic properties. To exploit these beneficial effects, fish oil was encapsulated and delivered to prevent capsular contracture. However, fish oil, being lipophilic, is incompatible with aqueous living systems and requires modification. Therefore, we modified it into a nanoemulsion formulation via low-energy phase inversion composition (PIC), which results in the formation of an o/w emulsion^45^. To solubilize fish oil in water, surfactants were first added. We used two small-molecule non-ionic surfactants, TWEEN 80 and SPAN 80, which can form small nanoparticles and are non-toxic^46^. A mixture of TWEEN 80 and SPAN 80 was added to the oil phase during emulsification (Fig. 1a). TWEEN 80 is a hydrophilic surfactant with an HLB value of 4.3 and SPAN 80 is a hydrophobic surfactant with an HLB value of 15. A mixture of these two surfactants enabled the emulsification of fish oil into an o/w nanoemulsion with small oil droplets. A TWEEN 80 to SPAN 80 ratio of 4:1 generated a nanoemulsion with very small particles and a low PDI (Fig. S1).

The addition of PEG 400 to the nanoemulsion minimized the oil droplet size and maximized stability (Fig. 1b). As shown in Fig. S2, the optimum weight ratio was 3.4%; higher or lower weight ratios resulted in larger oil particles. This amount was henceforth used to prepare NE-ω3. The ionic and non-ionic surfactants stabilized the fish oil and facilitated emulsification. An absolute zeta potential value of less than –30 mV indicates good stability^47^. Following the same protocol, we prepared a formulation with identical components and another lacking PEG 400. The zeta potentials of the formulations were determined using a Zetasizer Nano ZS90 (Malvern Instruments Ltd., Korea). For the latter sample, the absolute zeta potential value was lower (–1.75 ± 0.1) than the former sample (–34 ± 1.21) (Fig. S4). Therefore, PEG 400 is required to generate a stable nanoemulsion.

A portion of the emulsion was added to a poloxamer mixture (PF127:PF68). They are amphiphilic nonionic triblock copolymers containing hydrophilic polyethylene oxide (PEO) and hydrophobic polypropylene oxide (PPO) regions. The PEO and PPO blocks in the poloxamer molecule form a micellar structure in water with hydrophobic regions at the core and hydrophilic regions at the end, which makes it a liquid at room temperature. As the temperature increases, the sol-to-gel transition occurs, and the micelles rearrange into an orderly gel network (Fig. 1c). The two-step preparation method ensures that fish oil is trapped within the hydrophobic cavities of the poloxamers.

Next, the N3G formulation was optimized in terms of particle size, PDI, and zeta potential to maximize the ω3 content in N3G. To determine the maximum amount of fish oil that could create a successful nanoemulsion, 10%, 13.8%, 20%, and 25% fish oil were emulsified according to our protocol. The quantities of the other components—surfactants (TWEEN 80, SPAN 80), co-surfactant (PEG 400), and DI water—were the standards (wt%). An aliquot of nanoemulsion was diluted prior to DLS. As shown in Table 2, the particle sizes of 10% and 13.8% fish oil-containing nanoemulsions differed slightly (Fig. S5). Nanoemulsions with 20% and 25% had large particle sizes, possibly because of agglomeration of small particles to large aggregates. Although the particle size in the 10% fish oil nanoemulsion was smaller than that in nanoemulsion containing 13.8% fish oil, the latter had a lower PDI value, indicating a more homogeneous particle size. Thus, 13.8% fish oil was used in subsequent experiments.

**Table 2:** Optimization of the amount of fish oil for nanoemulsion preparation.

Sample no	Amount of fish oil (%)	Particle size (nm)	PDI	Zeta potential (mV)
1	10	248 ± 16.72	0.60 ± 0.073	− 33.5 ± 0.65
2	13.8	286 ± 6.939	0.27 ± 0.002	− 34 ± 1.21
3	20	1059 ± 127.2	0.83 ± 0.163	− 41.4 ± 1.1
4	25	1347 ± 157.6	0.53 ± 0.254	− 41.6 ± 0.72

Values are means ± SDs (n = 3).

**Fig. 2:**
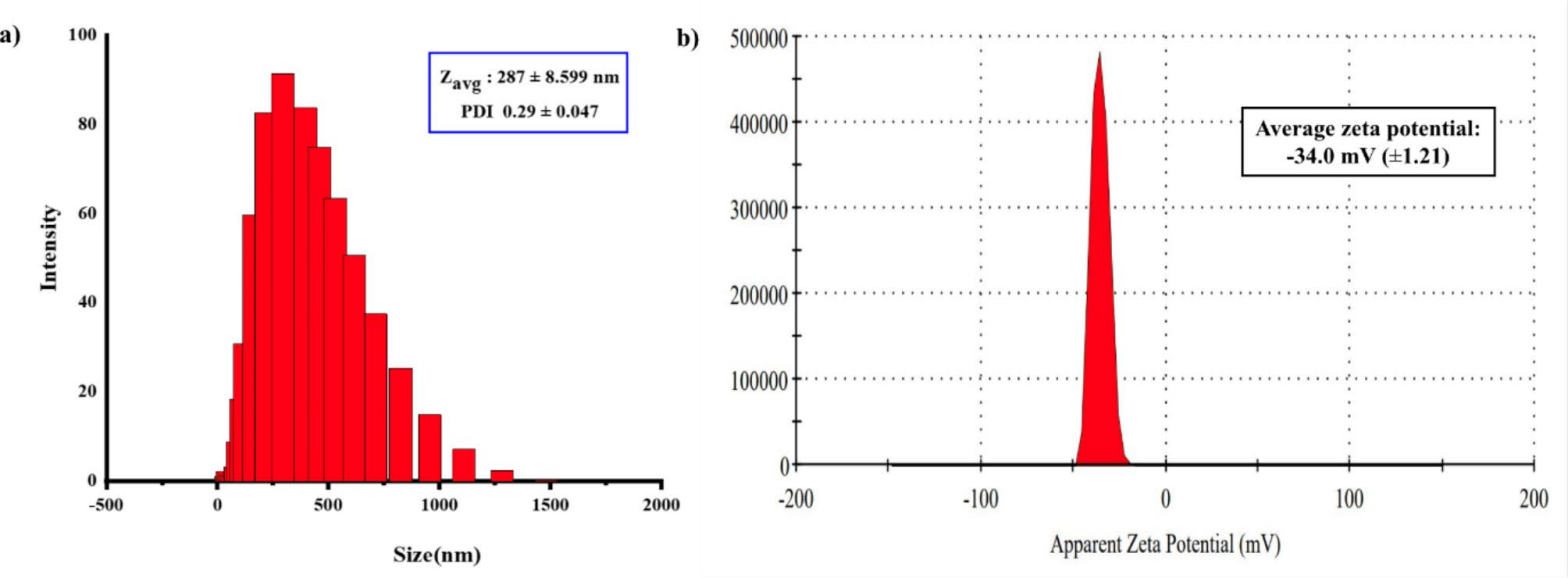
DLS analysis of the prepared nanoemulsion. (**a**) Particle size (Z_avg_) and PDI; (**b**) Zeta potential.

To determine the amount of fish oil needed to generate a visible nanoemulsion without layer separation, different formulations with identical volumes of DI water were investigated. As shown in Table S1, a maximum of 20 g fish oil could be added before the oil layer separated. The quantities (wt%) of fish oil, surfactant, and co-surfactant were as in the standard formulation. This formulation contained approximately 3 mg fish oil per 1 g gel. DLS showed that the oil droplet size was larger (Z_avg_ 5500 nm) than the standard formulation, and a macroemulsion was generated. Hence, this formulation was not considered further. The standard formulation (NE-ω3) contained 13.8% fish oil and had a very small particle size (mean 287 ± 8.599 nm) and a narrow particle size distribution (PDI 0.29 ± 0.047) according to DLS (Fig. 2a). The nanoemulsion had an average zeta potential of –34 ± 1.21 mV (Fig. 2b), indicating good physical stability and potential for drug delivery.

When surrounded by surfactants in a nanoemulsion, the activity of fish oil is preserved because of the absence of oxidation^48^. As shown in Fig. S5, there were no major changes in the Z_avg_, PDI, and zeta potential of NE-ω3, indicating preparation of a stable nanoemulsion.

After the preliminary stability study, we investigated the effects of temperature and pH on the stability of NE-ω3. Nanoemulsion samples were heated from room temperature to 37 °C. In addition, nanoemulsion samples were adjusted to acidic or basic, and the particle size, PDI, and zeta potential were evaluated. As shown in Table S2, the particle size increased by 9.78% and the PDI decreased by 8.31% after heating to 37 °C for 24 h. These changes may be attributable to Ostwald ripening, a common phenomenon in nanoemulsions^49^. In addition, the zeta potential of the acidic nanoemulsion was 99.661% lower than the neutral formulation, whereas that of the basic nanoemulsion was 47.3% higher. These results indicate that basic or acidic conditions can reduce the stability of NE-ω3.

NE-ω3 was converted into a thermoreversible gel. Thermoreversibility, which can be mediated by poloxamers, is the transition of a gel between liquid and semi-solid states in response to temperature changes and enables precise control of gel formation and drug release. Thermoreversibility is useful for implant coatings because the gel is liquid at room temperature but solidifies at a higher temperature (30–50 °C)^35,36^. PF127 was the primary gelling agent and formed a gel that encapsulated the fish oil micelles. However, this gel had a very low gelation temperature (< 20 °C). We added PF68 to the formulation to increase the gelation temperature. PF127 and PF68, at a variety of ratios, were added to 2 g diluted nanoemulsion and thermoreversibility was evaluated (Table 3). The addition of < 2 wt% PF68 resulted in thermoreversible N3G (Fig. S6). Solutions with PF127:PF68 ratios of 30:2 and 30:3 formed a gel at room temperature, but this gelation was disrupted by shaking. The optimum PF127: PF68 ratio for thermoreversible N3G was 30:1; this ratio was used in subsequent experiments. Gelation of N3G was optimal at 35 °C, indicating that it solidifies rapidly inside the body. The gelation time was ~ 5 min, enabling drug delivery to target locations.

**Table 3:** Determination of the optimum poloxamer ratio.

Poloxamer ratio (wt%)		Nature of the formulation	
PF127	PF68	At r.t	At 37 °C
17	5	Liquid	Liquid
17	10	Liquid	Liquid
20	0	Breakable gel	Gel
20	2	Liquid	Liquid
20	4	Liquid	Liquid
20	5	Liquid	Liquid
25	0	Gel	Gel
30*	1	Liquid	Gel
30	2	Liquid	Liquid
30	3	Liquid	Liquid

Pluronic F-127 (PF127); Pluronic F-68 (PF68). *Optimized ratio of poloxamers with which N3G is liquid at room temperature and a gel at 37 °C

The effects of heat and pH on the stability of N3G were investigated via FTIR. N3G was stable at 50 °C for 24 h. However, the C=O vibrational band (1743 cm^–1^) disappeared at pH 5 and 9 (Fig. S7), indicating that fish oil decomposes under acidic and basic conditions.

**Fig. 3:**
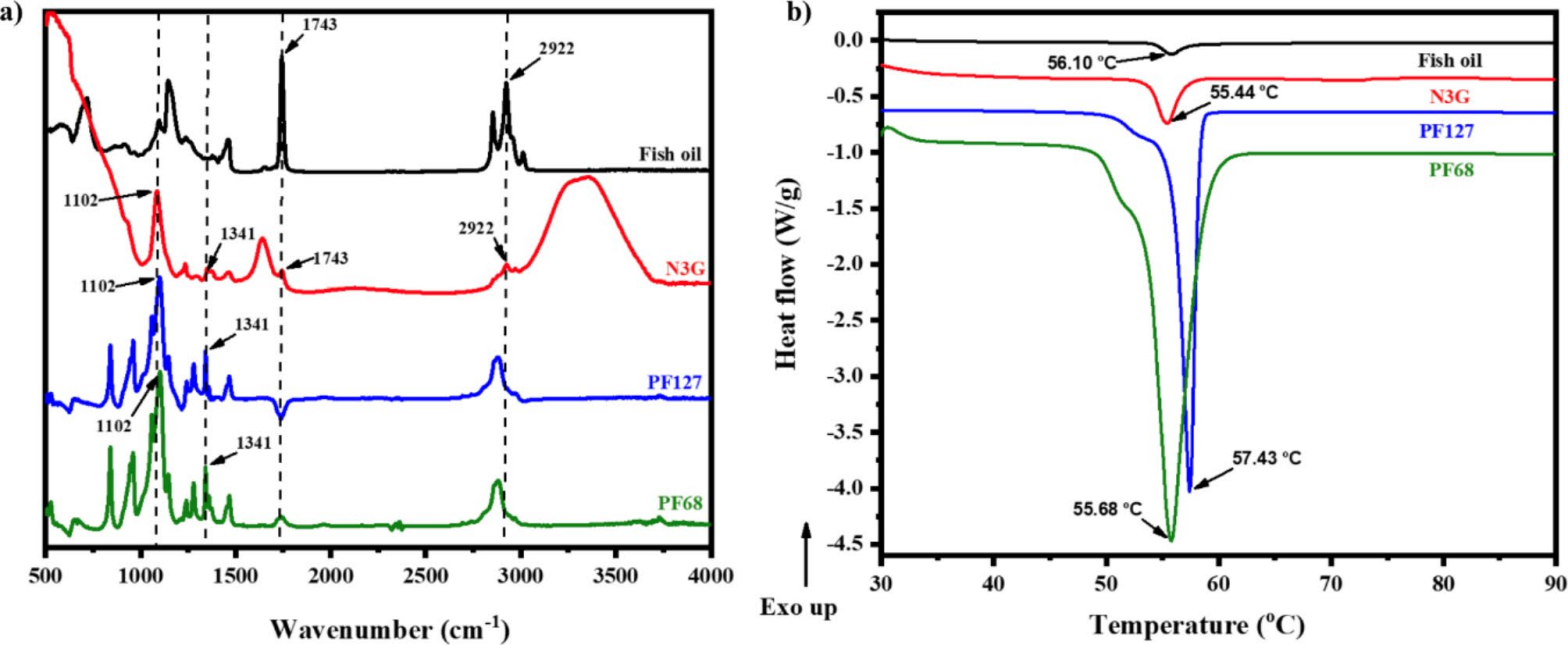
Characterization of N3G. (**a**) FTIR spectra of N3G and its precursor materials; (**b**) DSC spectra of N3G and its precursor materials.

N3G was characterized by FTIR spectroscopy to determine its chemical composition (Fig. 3a). The FTIR spectrum of N3G was compared to its precursor materials (fish oil and poloxamers). Fish oil showed major peaks at 2922 cm^–1^ and 1743 cm^–1^ due to the presence of C-H and C=O bonds. The major peaks for poloxamers were at 1341 cm^–1^ and 1102 cm^–1^, corresponding tο Ο–Η and C–O bonds, respectively. N3G showed peaks at 2922 cm^–1^, 1743 cm^–1^, 1341 cm^–1^, and 1102 cm^–1^, as did fish oil and poloxamers. These overlapping peaks confirm the presence of all of the components in N3G and that the components are physically blended, intact, and have unaltered chemical structures.

The thermal behavior of N3G was investigated via DSC. Figure 3b shows the DSC thermograms of N3G, fish oil, solid PF127, and solid PF68. The oil showed an endothermic peak at 56.10 °C due to the presence of saturated fatty acids such as myristic acid in fish oil^50^. PF68 and PF127 showed single sharp endothermic peaks at 55.68 °C and 57.43 °C, respectively, which are their melting points^51,52^. A single endothermic peak of N3G appeared at 55.44 °C during heating, suggesting that N3G has a semi-crystalline structure that melts at around 55.44 °C.

### In vitro drug release study of N3G

**Fig. 4:**
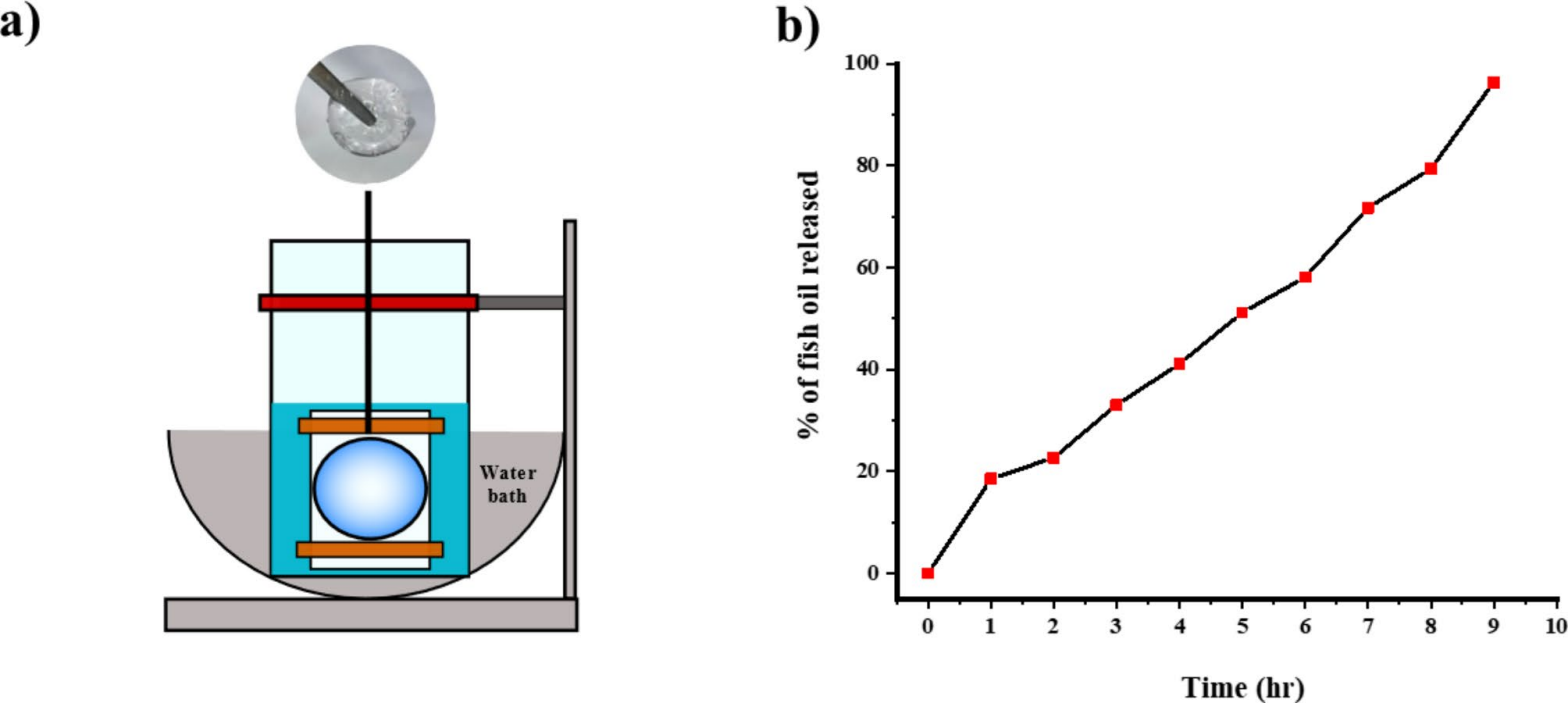
In vitro drug release by N3G. **(a**) Experimental setup to analyze drug release by N3G; (**b**) drug-release profile of fish oil. The amount of fish oil released over time is shown as a percentage.

In vitro drug release—i.e., the time required for the complete release of the encapsulated fish oil from the gel matrix—was investigated. A small silicone mini-implant was used to mimic the real-life situation. The surface of the mini-implant was coated with N3G and it was placed inside a perforated tube. The assembly was fully submerged in a beaker containing 50 mL DI water. The temperature was maintained at 37 °C using a water bath (Fig. 4a). Both ends of the tube were secured with weighted dialysis closures to ensure full immersion and inhibit leakage or floating.

Capsular contracture typically develops during the first few months after implantation; therefore, sustained drug release to the breast capsule is important at an early stage^2,53^. Compared to previous studies on poloxamer as a drug carrier, N3G exhibited sustained release^54,55^, and UV–vis absorption analysis showed that > 96% of the fish oil was released gradually over 10 h (Fig. 4b). The consistently slow release of fish oil from the DDS indicates that N3G can mediate controlled release, which is crucial in therapeutic contexts. Release could be slower in vivo (e.g., tissues and extracellular matrix), which differs markedly from the experimental conditions, underscoring the gel’s potential for clinical applications. N3G persistently adhered to the mini-implant surface, demonstrating the potential of N3G for biomedical applications, such as implant coatings, based on its sustained drug release.

### Animal study with implants and N3G

**Fig. 5:**
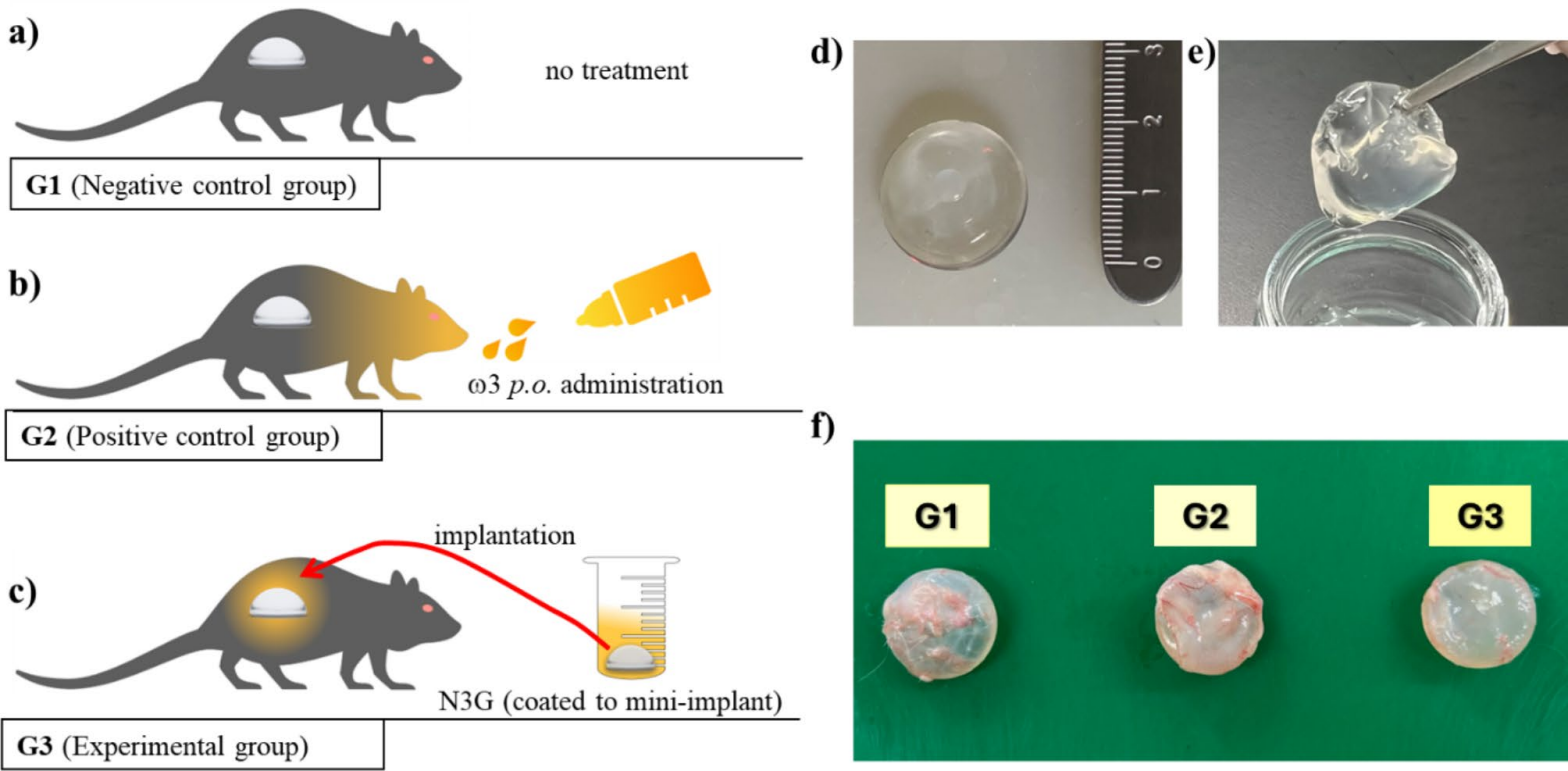
Study design. (**a**) G1, untreated negative control; (**b**) G2, positive control (treated daily with Menhaden fish oil [80 µL/250 g] via gavage); (**c**) G3, experimental group (N3G-coated implants [equivalent to a 0.6 g dose]); (**d**) custom-made, smooth, round silicone gel mini-implant (2 cc, 2 cm in diameter); e) mini-implant coated with N3G by immersion; f) implants and surrounding fibrous tissue (capsule).

To evaluate the practical utility of N3G, we performed an animal study using N3G-coated mini-implants (Fig. 5). Thirty rats were randomly assigned to three groups (n = 10 per group): G1 (negative control) received only the implants installed; G2 (positive control) received fish oil via gavage together with the installed implants; G3 (experimental group) received implants coated with N3G. Initially, the rats were implanted with a 2 cc custom-made, mini-implants of 2 cm diameter made of silicone gel (HansBiomed Co. Ltd., Seoul, Korea). Only rats in G3 received implants coated with N3G. All of the rats showed stable weight gain and food intake, suggesting the biocompatibility and non-toxicity of N3G. The study was terminated 90 days after the implantation surgery. The implants and surrounding capsules were excised as a single unit for use in subsequent experiments.

### Histological analyses of capsular tissues

**Fig. 6:**
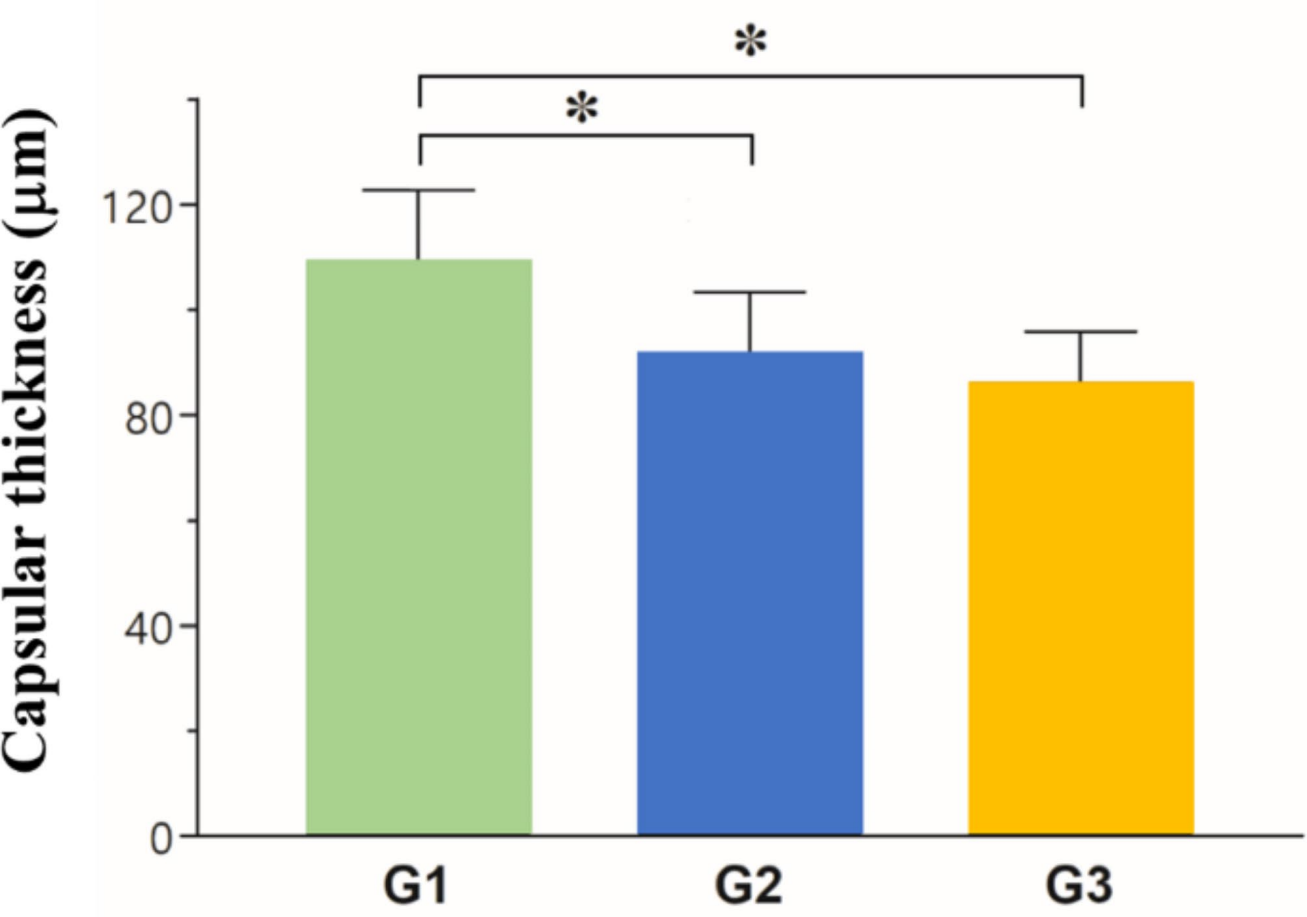
Average capsular thickness was 109.13 μm in G1, 91.52 μm in G2, and 86.00 μm in G3. Average capsular thickness differed significantly different between G1 and G2 according to post hoc analysis (*p < 0.05).

**Fig. 7:**
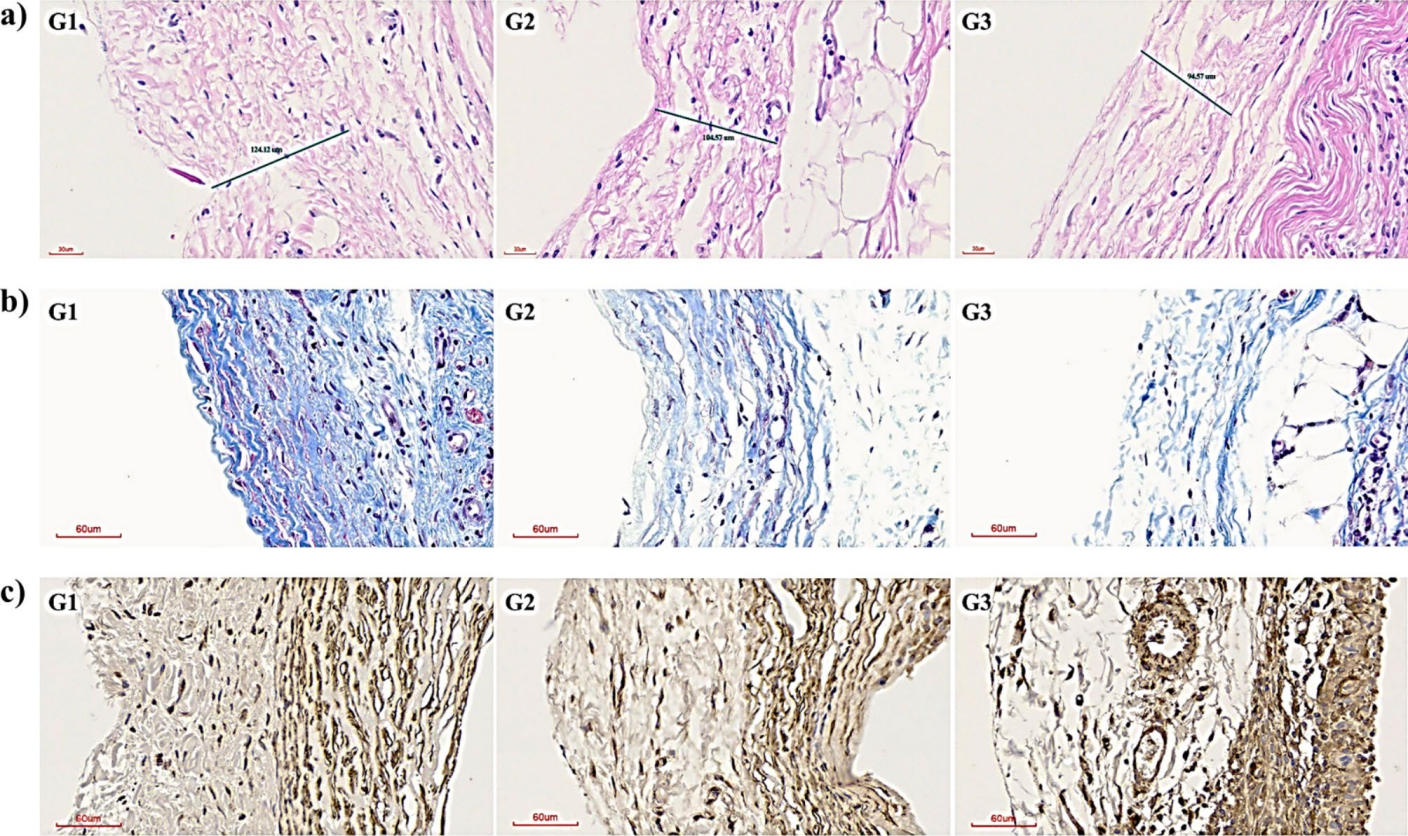
**(a**) H&E-stained sections of capsules from in G1 (left), G2 (center), and G3 (right); scale bar, 30 µm. (**b**) MT-stained sections showing fibrosis and collagen in G1 (left), G2 (center), and G3 (right); scale bar, 60 µm. (**c**) IHC sections stained with an anti-α-smooth muscle actin (α-SMA) antibody showing the myofibroblast distribution in G1 (left), G2 (center), and G3 (right); scale bar, 60 µm.

Excised capsular tissue was subjected to H&E staining to measure capsular thickness. Capsular thickness was decreased around the implants (G1, 109.13 ± 13.58 μm; G2, 91.52 ± 11.78 μm; G3, 86.00 ± 9.90 μm) (Figs. 6 and 7a). ANOVA indicated a significant reduction in G2 compared to G1; this finding was substantiated through a Fisher’s PLSD test, which showed significantly reduced thickness in both G2 and G3 compared to G1, with no marked difference between G2 and G3. MT staining was performed to identify fibrosis and collagen around the mini-implant. It revealed a progressive decrease in fibrosis and collagen levels in all groups, which was most notable in G3 (Fig. 7b). IHC indicated a considerable decline in myofibroblasts, which are vital for the development of fibrosis, with G1 and G3 presenting the highest and lowest counts, respectively (Fig. 7c). Taken together, these results indicate that N3G reduces capsular thickness, fibrosis, and collagen deposition.

The lack of a significant difference in capsular thickness between G2 and G3 may be attributable to the oral administration of fish oil in G2 for 90 days. Although capsular thickness did not significantly differ among the groups, MT staining of fibrosis and collagen and IHC of myofibroblasts suggested that G3 showed greater reductions of fibrosis and myofibroblast counts around the implant site than the other two groups. Based on this and the potential challenges of long-term compliance with oral supplementation, the N3G formulation is the most feasible for preventing capsular contracture.

### Gene expression analysis

The formation of thick fibrous scar tissue around a breast implant is a symptom of capsular contracture. Fibroblasts trigger fibrosis, which transform into myofibroblasts. Myofibroblasts form mesh-like scar tissue with blood vessels to seal the wound. Fibroblasts are responsive to inflammatory cytokines, such as COL1A2, TGF-β2, IFN-γ, IL-4, IL-6, and IL-10^56,57^. In qRT-PCR, extracted RNA is converted into complementary DNA (cDNA) by reverse transcription (RT). The cDNA is amplified and quantified by real-time PCR. During the RT step, a set of primers complementary to the mRNA of the gene of interest was used to specifically amplify the cDNA in the next step^58^.

**Fig. 8:**
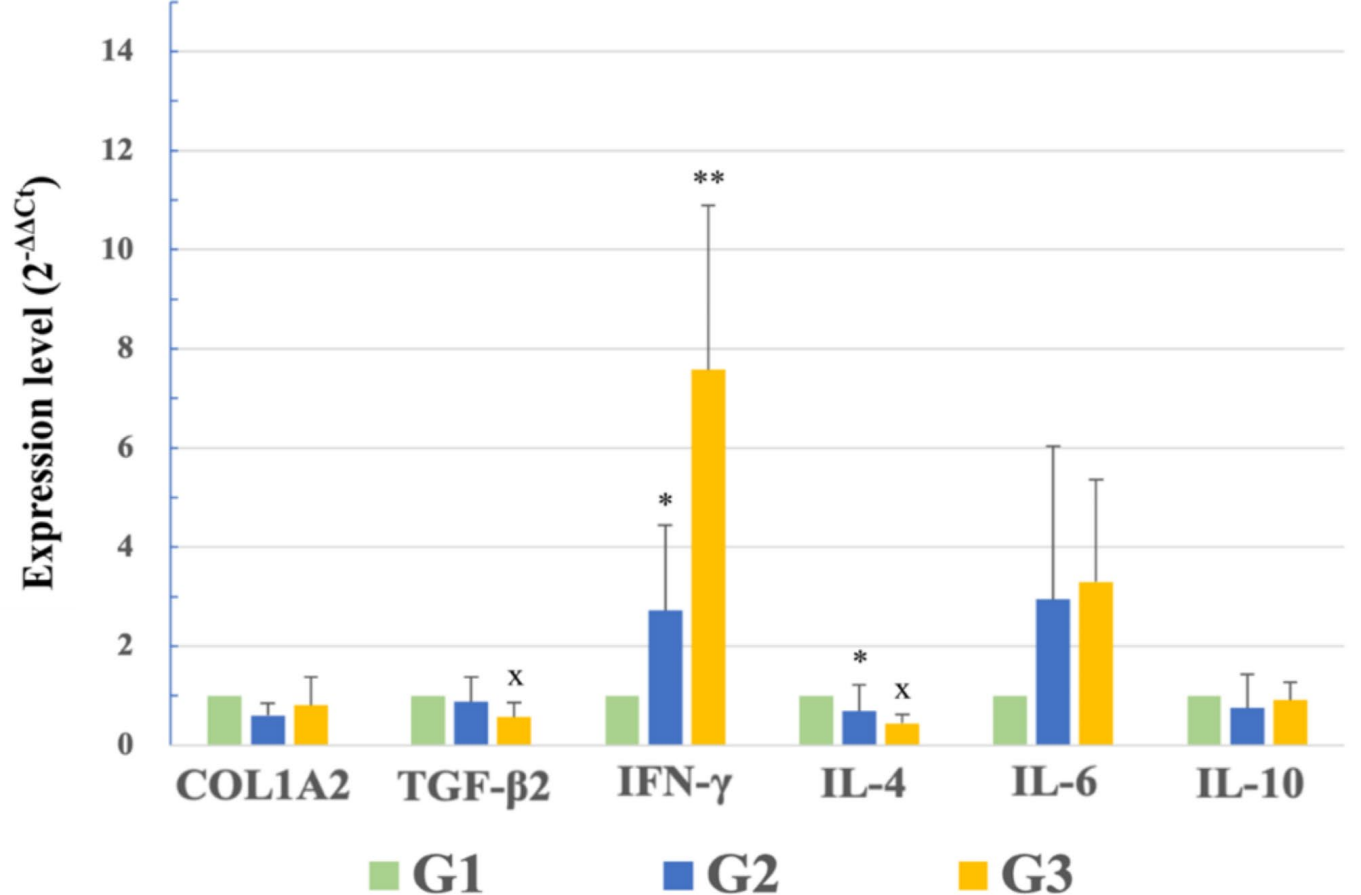
qrt-PCR analysis of the expression in capsule tissues of collagen alpha-2(I) chain (COL1A2), transforming growth factor (TGF)-β2, interferon (IFN)-γ, interleukin (IL)-4, IL-6, and IL-10. IFN-γ expression was significantly higher in G3 than G2 and G1, and IL-4 expression was lower in G3 and G2 than in G1. TGF-β2 expression was significantly lower in G3 than in G1. Data are means ± standard error of the mean. Significantly different from G1 vs. G2 (ANOVA post hoc test: *p < 0.05), G1 vs. G3 (ANOVA post hoc test: x, p < 0.05) and G2 vs. G3 (ANOVA post hoc test: **p < 0.001).

The expression levels of inflammatory cytokines in capsule tissues are shown in Fig. 8. The expression of IFN-γ was significantly higher in G3 than G2 and G1 (7.582 ± 3.318, 2.719 ± 1.730, and 1.000 ± 0.000, respectively). IFN-γ plays a complex role in the development of capsular fibrosis around implants. It modulates the immune response, activates macrophages, and inhibits fibroblast activity, which are crucial for collagen production and extracellular matrix formation. It also regulates other inflammatory cytokines, thereby affecting fibrosis progression. It reduces myofibroblast viability and increases apoptosis, leading to decreased collagen formation and fibrosis^59–61^. The highest IFN-γ level in G3 is consistent with the antifibrotic property of ω3 and suggests that N3G has potential for preventing capsular contracture. IL-4 expression was significantly lower in G3 (0.444 ± 0.178) and G2 (0.689 ± 0.528) than G1 (1.000 ± 0.000), indicating lower IL-4 levels in G3 and G2 compared to G1. This reduction indicated a decrease in the inflammatory response, consistent with the regulatory role of IL-4 in M2 macrophage polarization and inflammation. This reduction might indicate less need for the anti-inflammatory and tissue remodeling functions typically mediated by IL-4. Given that M2 macrophages play a role in healing and anti-inflammatory processes, lower IL-4 expression may reflect an altered inflammatory response or reduced inflammation in the context of capsular contracture^2,62^.

TGF-β2 expression was significantly lower in G3 (0.569 ± 0.291) than in G1 (1.000 ± 0.000), with G2 showing a value of 0.874 ± 0.501. This significant reduction in TGF-β2 expression in G3 emphasizes the importance of TGF-β in fibrosis, particularly in the context of chronic inflammation. This reduction is particularly notable, given the involvement of TGF-β in collagen deposition and fibrosis during the foreign body response, which is often triggered during processes occurring in the acute phase of inflammation, such as platelet degranulation. This decrease in TGF-β expression aligns with previous reports that its suppression not only reduces scar formation but also myofibroblast contraction. Therefore, the lower TGF-β2 level in the treatment group indicate the effectiveness of the gel in mitigating fibrotic reactions around implants^63–66^. The expression levels of COL1A2, IL-6, and IL-10 were not significantly different among the three groups. COL1A2 expression was 0.811 ± 0.566 in G3, 0.605 ± 0.243 in G2, and 1.000 ± 0.000 in G1. IL-6 expression was 3.300 ± 2.055 in G3, 2.944 ± 3.096 in G2, and 1.000 ± 0.000 in G1. IL-10 expression was 0.918 ± 0.355 in G3, 0.758 ± 0.670 in G2, and 1.000 ± 0.000 in G1.

### Mechanistic insight into ω3 PUFAs in inflammation and fibrosis

Ω3 PUFAs have anti-inflammatory and antifibrotic properties, which involve multiple biochemical pathways and cellular processes. They reduce inflammation and fibrosis by serving as precursors to specialized pro-resolving mediators such as resolvins, which inhibit pro-inflammatory eicosanoid production and cytokine expression, thus dampening the inflammatory response. In addition, **ω**-3 PUFAs compete with **ω**-6 fatty acids, displacing arachidonic acid and reducing the synthesis of inflammatory compounds^67,68^. In fibrosis, **ω**-3 PUFAs promote the degradation of Yes-associated protein and transcriptional co-activator with PDZ-binding motif in the liver and kidneys. This reduces hepatic stellate cell activation and enhances autophagy flux and AMP-activated protein kinase activation, thereby mitigating fibrosis^30,69^. They also upregulate nuclear factor erythroid 2-related factor 2, decreasing oxidative stress and apoptosis^70^. These mechanisms underline the therapeutic potential of omega-3 PUFAs in non-alcoholic steatohepatitis and rheumatoid arthritis, effects mediated by improvement of inflammation and tissue repair^71,72^.

### Effectiveness of N3G for capsular contracture

The strength of this study is its comprehensive assessment of N3G for the prevention of capsular contracture. The detailed histological and gene expression analyses provide insight into the anti-inflammatory and antifibrotic effects of ω3 PUFAs. The use of an established rat model, with appropriate control groups, enhanced the reliability and validity of the findings. Moreover, the thermoreversible gel facilitated sustained drug release and localized treatment, highlighting its potential as a safer and more effective alternative to current therapies.

N3G has several advantages over existing treatments for capsular contracture, including significant reductions in capsular thickness, fibrosis, and myofibroblast count, comparable to pharmacological options such as LTRAs and steroids, but with fewer side effects. Furthermore, it could improve patient compliance and reduce the frequency of treatments. In addition, by preventing capsular contracture, N3G could reduce the need for costly revision surgeries, thereby minimizing the financial burden on patients and healthcare systems. These attributes underscore the innovative nature and clinical relevance of N3G.

## Conclusion

Our findings provide insight into the ability of N3G to prevent capsular contracture after breast-implant surgery. We encapsulated fish oil in the hydrophobic cavity of micelles and added gelation agents to generate a nanoemulsion-based gel. The gel contained the optimum amount of fish oil and showed thermoreversibility. In vivo assays showed significant reductions in capsular thickness, fibrosis, and myofibroblast counts in N3G-treated rats, indicating delivery of ω3 PUFA into the mini-implants. The findings demonstrate the efficacy of N3G and the importance of ω3 PUFAs in suppressing fibrosis. The ability to overcome challenges related to dosing and storage further underscores the potential of N3G as a reliable treatment option for capsular contracture. Nevertheless, the 90-day duration of the experiment hampered evaluation of the long-term effect of N3G, and longer-duration studies are underway.

### Ethics statement

Animals were housed in pairs under standard conditions and had free access to food and water. Environmental enrichment was provided, and steps were taken to minimize suffering. Isoflurane anesthesia was used for surgery. Thirty rats were divided into three groups, with 10 rats per group. Euthanasia was performed via carbon dioxide inhalation followed by cervical dislocation. We confirm that the experimental procedures described in this manuscript adhere to the Animal Research: Reporting of In Vivo Experiments (ARRIVE) guidelines. Animal procedures adhered to the guidelines of the Woojung Center for Phenogenomics and were approved by its Institutional Animal Care and Use Committee (Approval No. IACUC2302–012; date of approval April 26 2023).

## Electronic supplementary material

Below is the link to the electronic supplementary material.

Supplementary Material 1

## Data Availability

The authors confirm that the data supporting the findings of this study are available within the article and its supplementary materials.
