# Supplementary material for: Development of a fish oil–nanoemulsion gel as a drug-delivery system to prevent capsular contracture
Source: Sci Rep. 2024 Nov 26;14:29385. doi: 10.1038/s41598-024-81122-6 (PMC11599770; doi:10.1038/s41598-024-81122-6)
Supplement: Supplementary file 1 — Supplementary Material 1 [file 41598_2024_81122_MOESM1_ESM.docx]

**Supplementary materials**

**Development of Fish Oil-Nanoemulsion Based Gel as a Drug Delivery System to Prevent Capsular Contracture**

Mohuya Paul^a^, Sang Gue Kang^b^, Jungkyun Im^a,c*^, Woo Jin Song^b*^

*^a^Department of Electronic Materials, Devices, and Equipment Engineering, Soonchunhyang University, Asan 31538, Republic of Korea*

*^b^Department of Plastic and Reconstructive Surgery, Soonchunhyang University College of Medicine, Seoul, 04401, Republic of Korea*

*^c^Department of Chemical Engineering, Soonchunhyang University, Asan 31538, Republic of Korea*

*Correspondence should be addressed to Prof. Jungkyun Im; [jkim5279@sch.ac.kr](mailto:jkim5279@sch.ac.kr) and Dr. Woo Jin Song; [pswjsong@gmail.com](mailto:pswjsong@gmail.com)


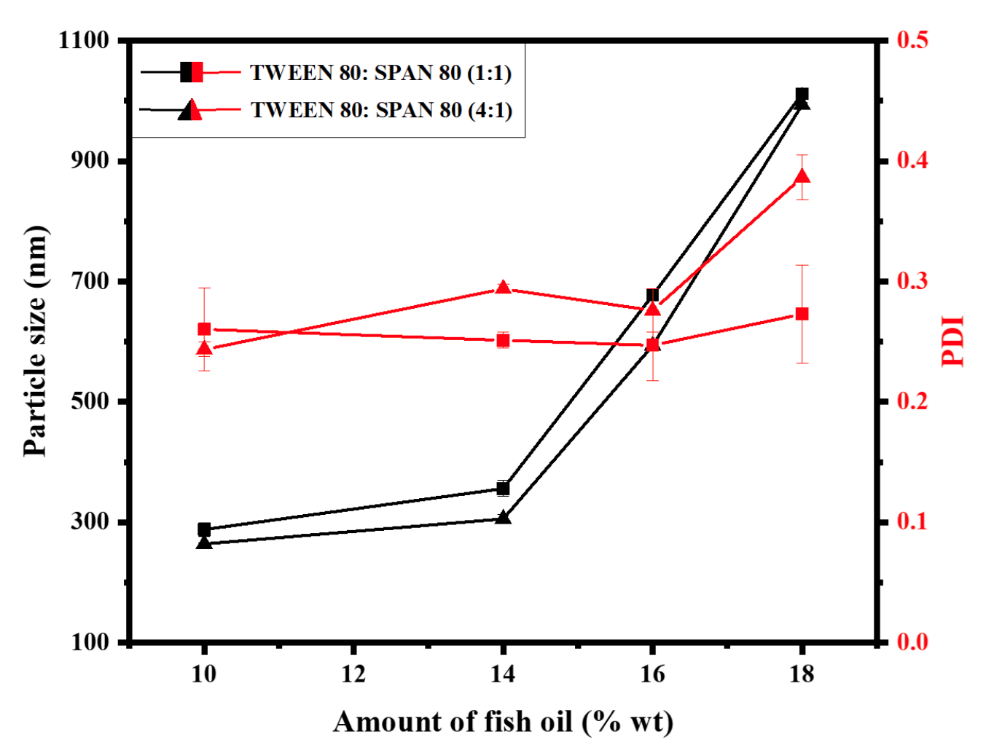


**Figure S1.** Effect of particle size and PDI on various non-ionic surfactant (TWEEN 80: SPAN 80) ratio.


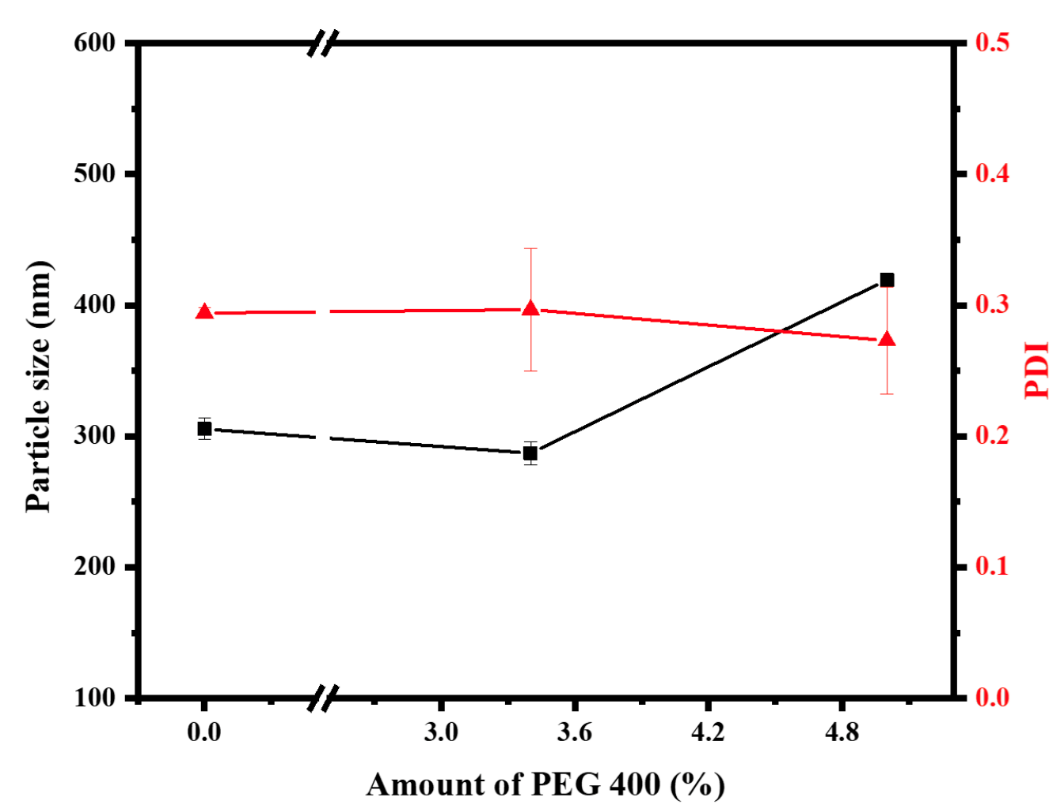


**Figure S2.** Effect of particle size and PDI on various co-surfactant (PEG 400) amount.


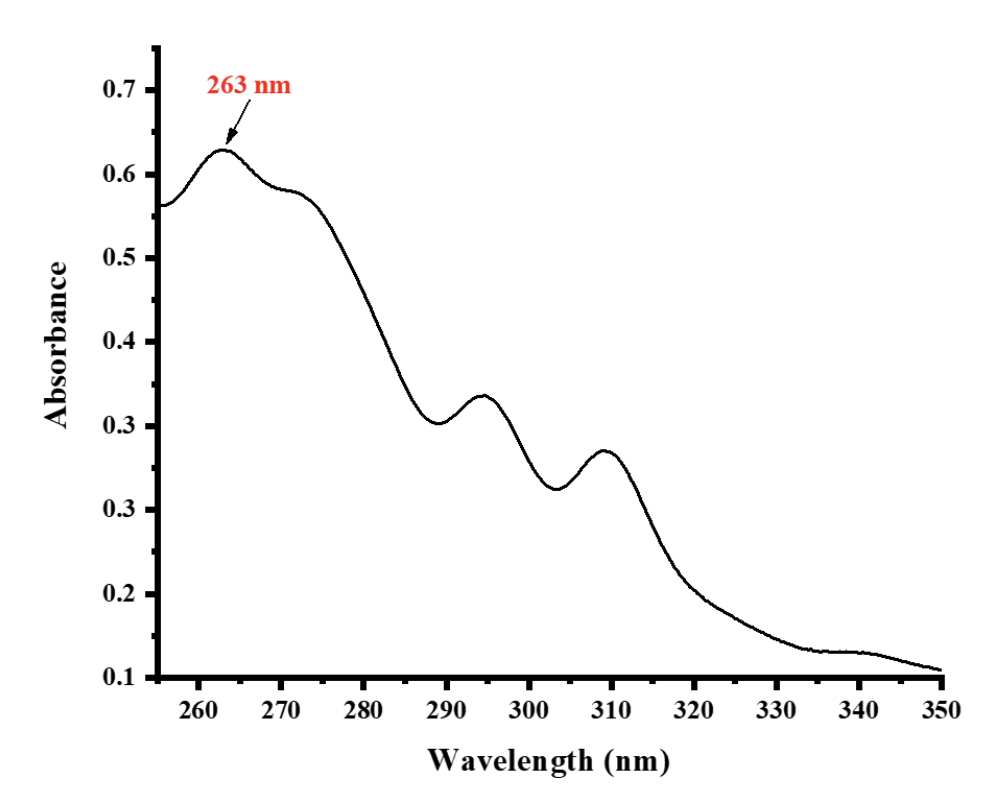


**Figure S3.** UV-Vis spectrum of fish oil in EA.


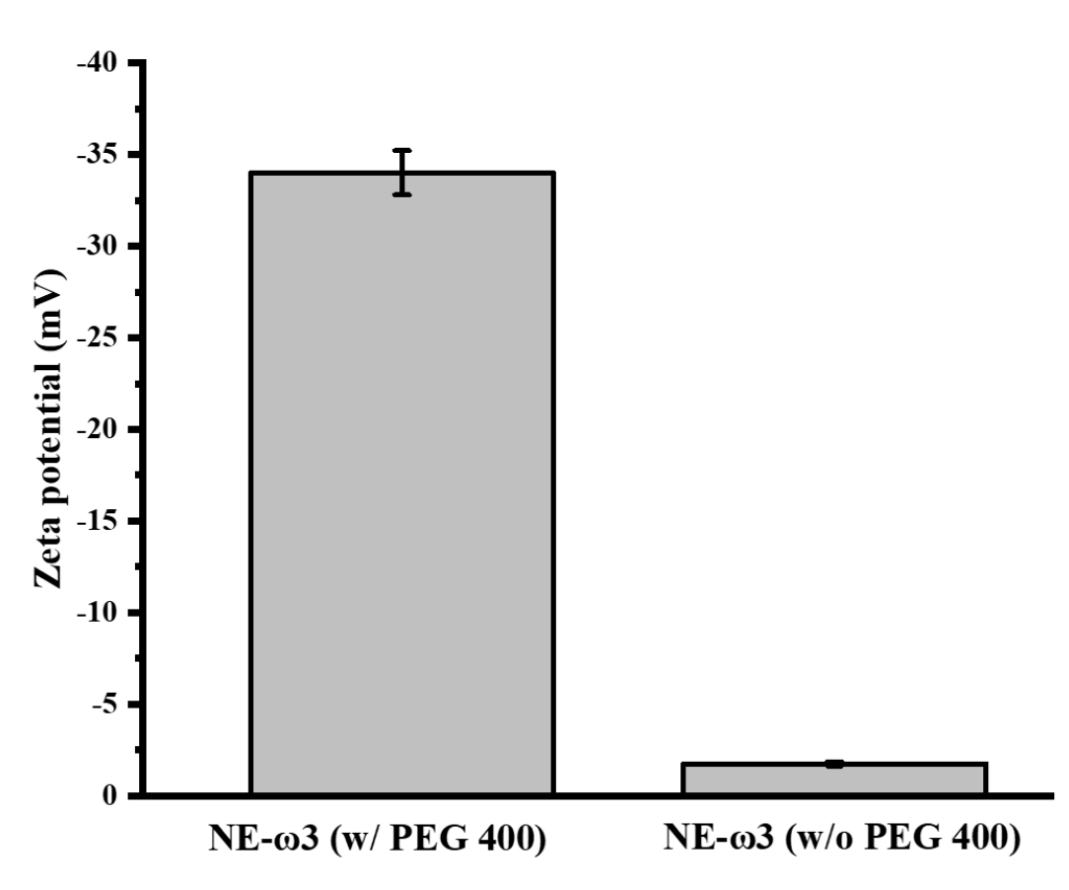


**Figure S4.** Comparison of the zeta potential values of nanoemulsion w/ and w/o PEG 400.


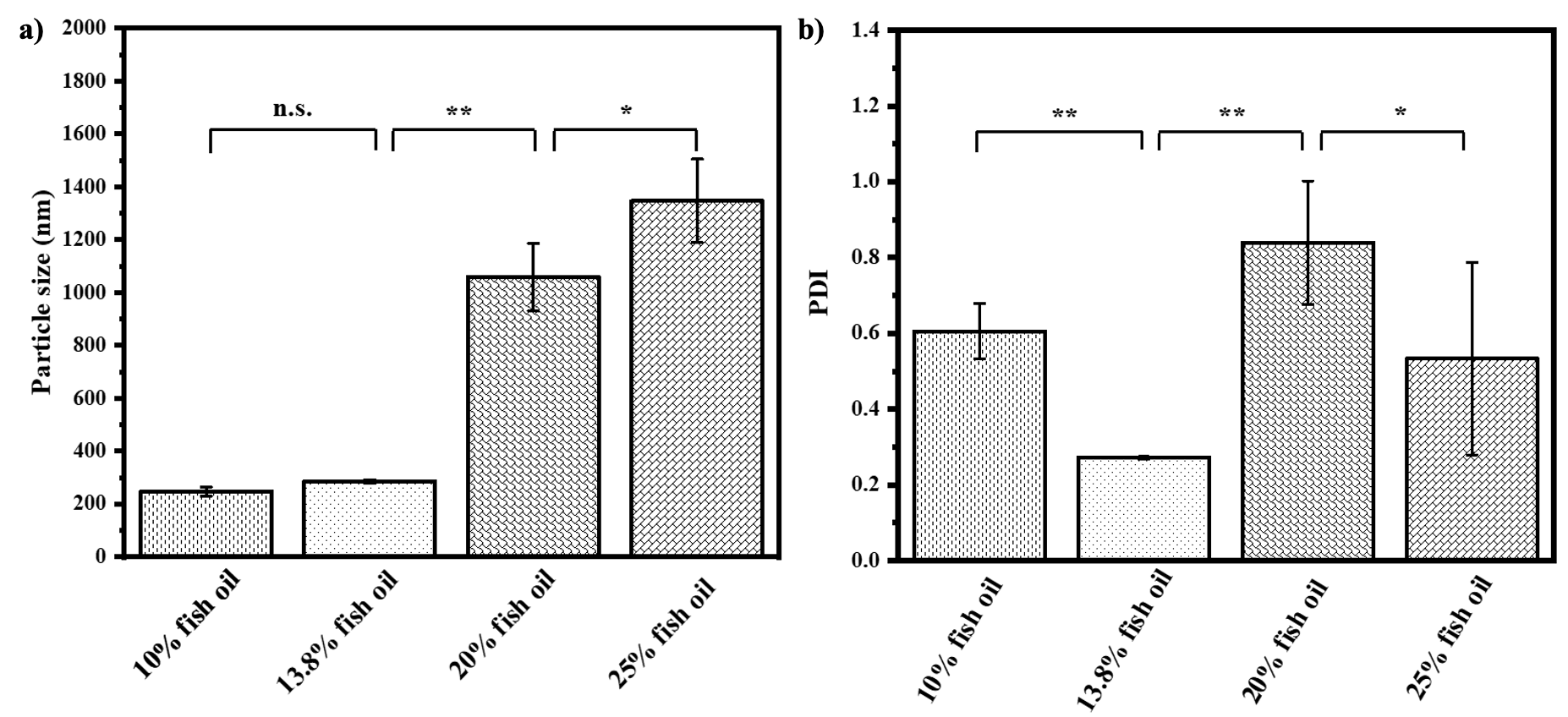


**Figure S5.** Comparison between a) particle size, and b) PDI value of nanoemulsion sample containing 10%, 13.8%, 20%, and 25% fish oil. Values are expressed as mean ± standard deviation. n.s. denotes non-significant differences between two groups. A single asterisk (*) indicates p < 0.05, and two asterisks (**) indicate p < 0.01.


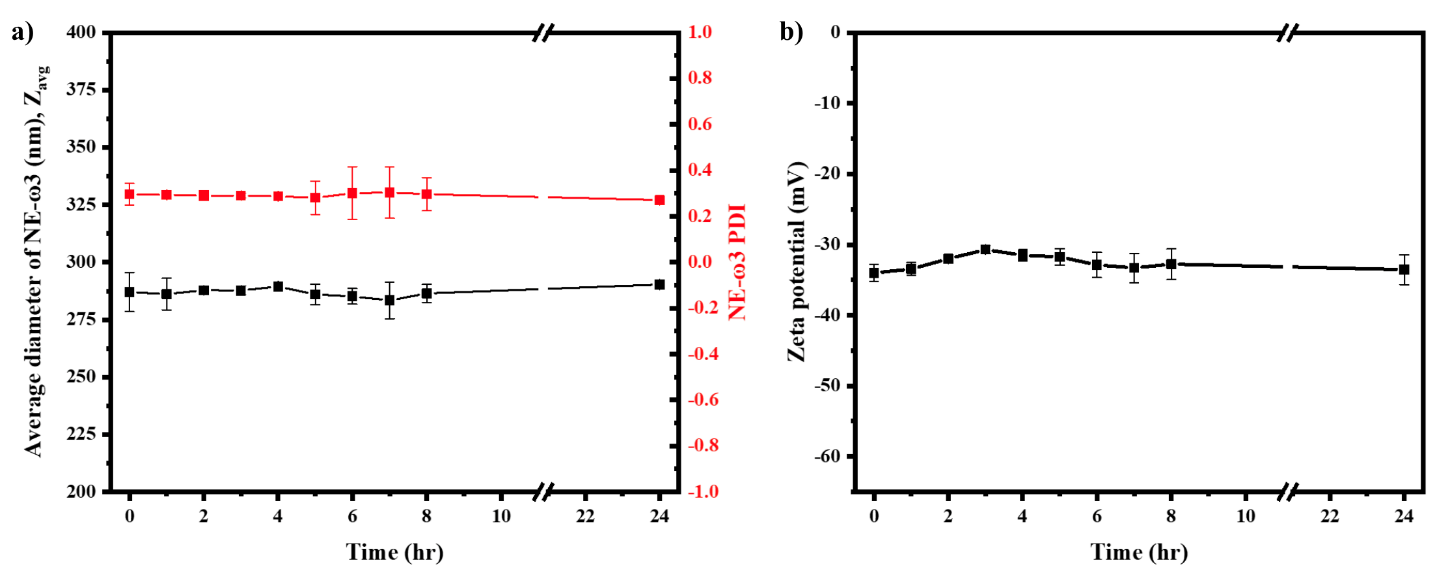


**Figure S6.** Stability study of NE-ω3 over time. a) changes in the Z_avg_ and PDI of NE-ω3 over time; b) changes in the zeta potential of NE-ω3 over time.


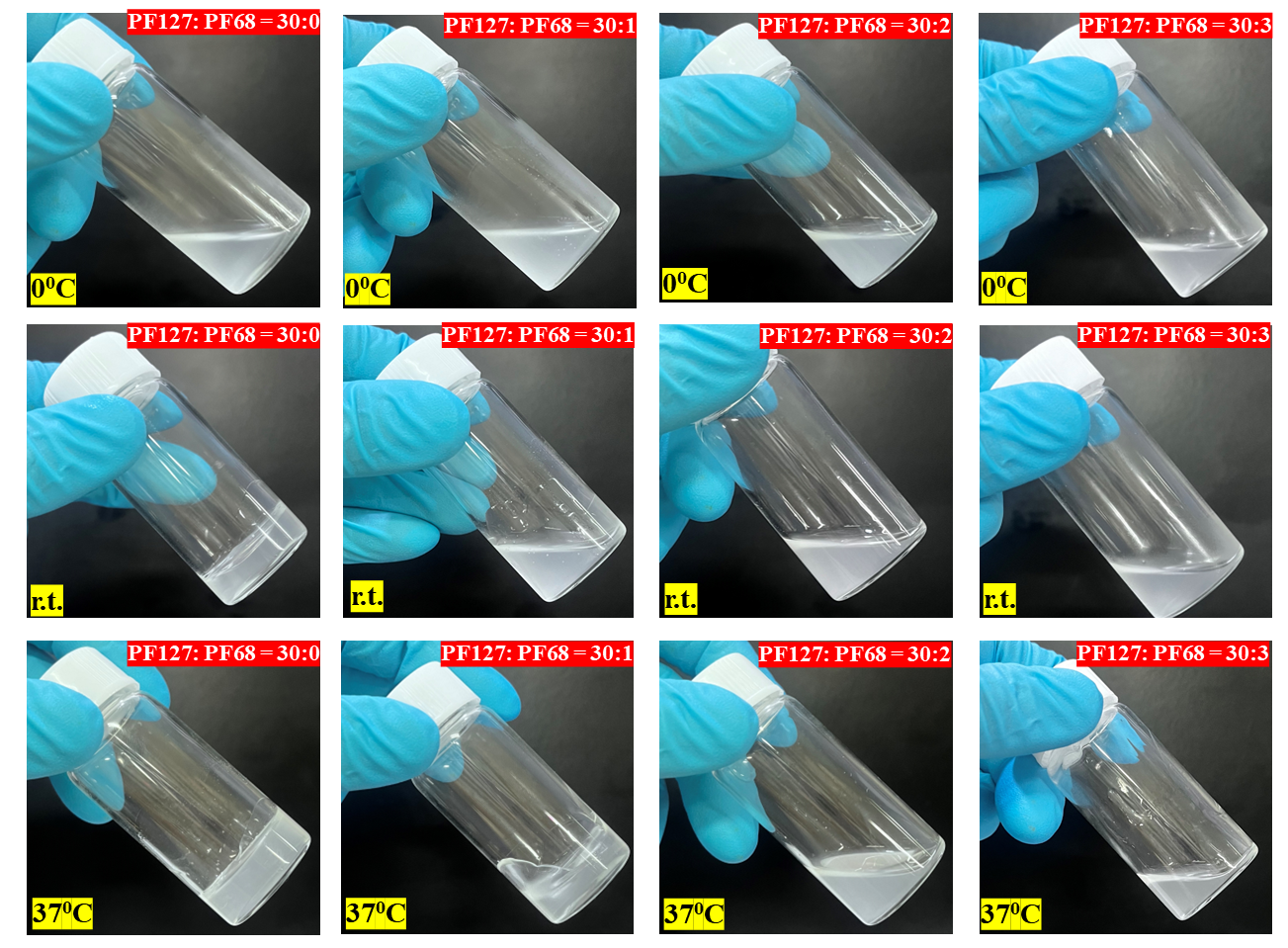


**Figure S7.** Observation of gelation at different temperature with varying amount of PF68 gel formulation. For every group, the amount of PF127 was kept constant (30% w/v).


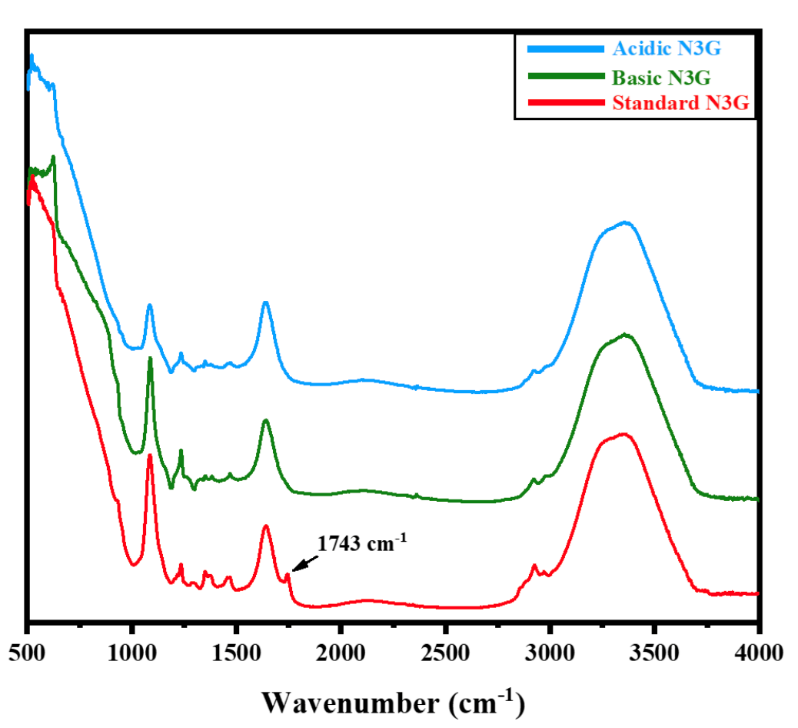


**Figure S8.** Comparison of FT-IR spectra of standard N3G, and N3G in acidic or basic conditions.

**Table S1.** Optimal fish oil concentration for nanoemulsion formulation.

| **Fish oil (g)** | **Fish oil (mg)/1g gel** | **Remarks** |
| --- | --- | --- |
| 1 | 0.53 | + |
| 5 | 1.76 | + |
| 10 | 2.40 | + |
| 15 | 2.80 | + |
| 20* | 3.02 | + |
| 25 | 3.17 | - |

*Indicates the maximum amount of fish oil that can be used to successfully make the nanoemulsion, + and – denote the successful and unsuccessful formation of nanoemulsion respectively.

**Table S2.** Effect of environmental factors on the stability of NE-ω3.

| **Temperature (^o^C)** | **pH** | **Particle size (nm)** | **PDI** | **Zeta potential (mV)** |
| --- | --- | --- | --- | --- |
| r.t. | - | 287 ± 8.599 | 0.29 ± 0.047 | -34.0 ± 1.21 |
| 37 | - | 315 ± 6.057 | 0.27 ± 0.002 | -36.2 ± 0.75 |
| r.t. | 5 | 278 ± 6.940 | 0.33 ± 0.112 | -0.11 ± 1.011 |
| r.t. | 9 | 278 ± 4.701 | 0.35 ± 0.079 | -50.1 ± 0.3 |

Values represented as mean ± SD (n=3); ‘-’ denotes that the parameter was kept unchanged during the experiment
